# Inhibition studies of *Brucella suis* β-carbonic anhydrases with a series of 4-substituted pyridine-3-sulphonamides

**DOI:** 10.1080/14756366.2017.1413097

**Published:** 2017-12-22

**Authors:** Simona Maria Monti, Angela Meccariello, Mariangela Ceruso, Krzysztof Szafrański, Jarosław Sławiński, Claudiu T. Supuran

**Affiliations:** a Institute of Biostructures and Bioimaging, Naples, Italy;; b Neurofarba Department, Section of Pharmaceutical Sciences, Università degli Studi di Firenze, Sesto Fiorentino, Florence, Italy;; c Department of Organic Chemistry, Medical University of Gdansk, Gdańsk, Poland

**Keywords:** Carbonic anhydrase, Brucella suis, sulphonamide, heterocycle, antibacterials

## Abstract

The two β-carbonic anhydrases (CAs, EC 4.2.1.1) from the pathogenic bacterium *Brucella suis*, BsuCA1 and BsuCA2, were investigated for their inhibition profile with a series of pyridine-3-sulphonamide derivatives incorporating 4-hetaryl moieties. BsuCA1 was effectively inhibited by these sulphonamides with inhibition constants ranging between 34 and 624 nM. BsuCA2 was less sensitive to these inhibitors, with K_I_s in the range of 62 nM - > 10 µM. The nature of the 4-substituent present on the pyridine ring was the main factor influencing the inhibitory profile against both isoforms, with 4-halogenophenylpiperazin-1-yl and 3,4,5-trisubstituted-pyrazol-1-yl derivatives showing the most effective inhibition. Some of these sulphonamides were most effective bacterial CA than human (h) CA I and II inhibitors, making them selective for the prokaryotic enzymes. Investigation of bacterial CA inhibitors may be relevant for finding antibiotics with a new mechanism of action compared to the clinically used agents for which substantial drug resistance emerged.

## 1. Introduction

Bacteria encode for at least three genetic families of the metalloenzyme carbonic anhydrase (CA, EC 4.2.1.1), the α-, β- and γ-CAs^1–3^. By catalysing the interconversion between carbon dioxide and bicarbonate, with formation or consumption of a hydronium ion, these widespread enzymes are involved in a multitude of physiologic processes connected with the pH regulation, biosynthetic processes in which CO_2_/bicarbonate are involved, photosynthesis, acclimation in various environments were the bacteria thrive, colonisation of the host and more^1–5^. Since CA inhibition from vertebrates, more exactly humans, in which 15 different α-CA isoforms were described^6^, has pharmacologic applications, the idea to exploit bacterial/microbial CA inhibition for obtaining antiinfectives with a new mechanism of action started to be explored in recent years^1–4^^,^^7–11^. Indeed, many classes of inhibitors for all three types of bacterial CAs were discovered to date, among which the sulphonamides represent the most investigated chemotype^12–16^. CA inhibitors (CAIs) targeting human enzymes (hCAs) are in clinical use for decades for the management of various diseases among which glaucoma, obesity, epilepsy, intracranial hypertension and as diuretics^13–16^. More recently they started to be used for the treatment of hypoxic tumours^13^^,^^14^, being also investigated as possible drugs for neuropathic pain^17^, cerebral ischemia^18^ and arthritis^19^.

*Brucella suis* is one of the bacteria responsible of brucellosis, a disease affecting an increasing number of people and which showed variable degrees of resistance to the clinically used antibiotics [1c,9–11]. Two β-class CAs were discovered in the genome of this pathogen, BsuCA1 and BsuCA2^9^^,^^10^, which have also been investigated for their inhibition with various compounds, such as sulphonamides, sulphamates, anions, phenols, etc.^9–12^. Furthermore, the growth of the bacterium was also impaired (in cell cultures) by some of these inhibitors, which constitutes the proof-of-concept that BsuCA1/2 inhibition may have a significant antibacterial effect^9^. Continuing our interest in the discovery of CAIs which effectively target bacterial CAs, we report here an inhibition study of BsuCA1/2 with a class of pyridine-3-sulphonamide derivatives incorporating 4-heterocyclic/heteroaryl moieties, previously designed by our groups for targeting the tumor-associated human isoforms hCA IX and XII^12^. Some of the investigated sulphonamides from this article are among the most effective and isoform-selective BsuCA1 inhibitors ever reported.

## 2. Materials and methods

### 2.1. Chemistry

Sulfonamides **1–18** used in this study were reported earlier by our groups^12^ and were used without further purification. Acetazolamide (**AAZ**), buffers and other inorganic reagents were the highest purity available reagents from Sigma-Aldrich (Milan, Italy).

### 2.2. Cloning, expression and purification of BsuCA1and BsuCA2

cDNA encoding BsuCA1 and BsuCA2 (a kind gift of Prof J.Y. Winum from University of Montpellier, France) were PCR engineered to be cloned in pETM13 (a kind gift from EMBL, Heidelberg) expression vector. The resulting plasmids, pETM13-*bsuca1* and pETM13-*bsuca2*, were verified by appropriate digestion with restriction enzymes and sequencing. BsCA1 and BsCA2 were expressed in LB culture medium by induction with 1 mM IPTG in *Escherichia coli* BL21 (DE3) and BL21 (DE3) plusS cells, respectively. After 16hs incubation at 22 °C, cells were harvested by centrifugation, lysed and affinity purified onto a 1 ml His Trap FF column and subsequently on a Superdex 75 10/300 GL column (GE Healthcare). Purity level of BsCA1 and BsCA2 was assessed by LC-MS and SDS-PAGE.

### 2.3. Carbonic anhydrase inhibition

An Applied Photophysics stopped-flow instrument has been used for assaying the CA catalyzed CO_2_ hydration activity^20^. Phenol red (at a concentration of 0.2 mM) has been used as indicator, working at the absorbance maximum of 557 nm, with 20 mM TRIS (pH 8.3) as buffer and 20 mM NaClO_4_ (for maintaining constant the ionic strength), following the initial rates of the CA-catalyzed CO_2_ hydration reaction for a period of 10–100 s. The CO_2_ concentrations ranged from 1.7 to 17 mM for the determination of the kinetic parameters (by Lineweaver–Burk plots) and inhibition constants. For each inhibitor at least six traces of the initial 5–10% of the reaction have been used for determining the initial velocity. The uncatalyzed rates were determined in the same manner and subtracted from the total observed rates. Stock solutions of inhibitor (10 mM) were prepared in distilled-deionized water and dilutions up to 0.1 nM were done thereafter with the assay buffer. Inhibitor and enzyme solutions were preincubated together for 15 min at room temperature prior to assay, in order to allow for the formation of the E-I complex. The inhibition constants were obtained by non-linear least-squares methods using PRISM 6 and the Cheng–Prusoff equation, as reported earlier^21–25^, and represent the mean from at least three different determinations.

## 3. Results and discussion

Aromatic and heterocyclic sulphonamides were showed earlier by some of us to act as inhibitors of the two β-CAs from *B. suis*, with various degrees of efficacy^9^^,^^10^. Usually, the heterocyclic derivatives, such as acetazolamide (5-acetamido-1,3,4-thiadiazole-2-sulfonamide, **AAZ**) were among the best bacterial CA inhibitors, but their efficacy was much better for the human isoforms hCA I and II (highly abundant proteins found in the blood, GI tract and many other tissues^6^^,^^13^) which may lead to a series of side effects if such inhibitors should be used as anti-bacterials. Thus, exploration of structurally different sulphonamides may lead to the discovery of compounds with a better inhibitory profile and better selectivity for the bacterial over the human isoforms. Thus, in the present study, we investigated a series of pyridine-3-sulfonamide derivatives incorporating 4- heterocyclic/heteroaryl moieties **1–18**, reported earlier by our groups as effective tumor-associated human isoforms hCA IX and XII inhibitors^12^.

Inhibition data of hCA I and II (offtargets) as well as BsuCA1/2 with sulfonamides **1–18** and acetazolamide as standard inhibitor are shown in Tables 1 and 2.

**Table 1.** Inhibition of human (h) CA isoforms hCA I and II and bacterial (*Brucella suis*) enzymes BsuCA1 and BsuCA2 with sulfonamides **1–12**, by a stopped-flow CO_2_ hydrase assay^20^. Acetazolamide (**AAZ**) was used as standard inhibitor.
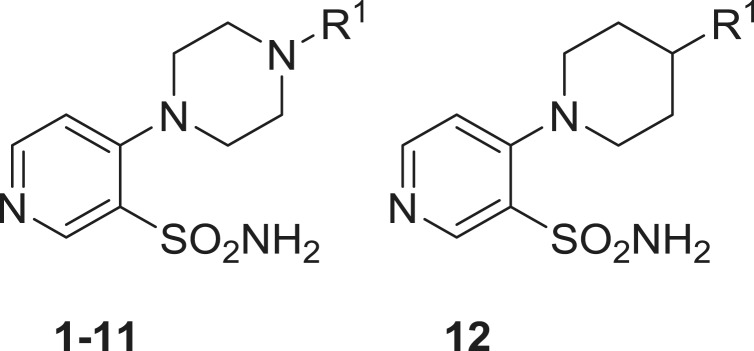

		K_I_ (nM)*			
No.	R^1^	hCA I^a^	hCA II^a^	BsuCA1^b^	BsuCA2^b^
**1**		3320	436	121	860
**2**		2450	389	61	62
**3**		1285	354	62	915
**4**		4335	295	346	822
**5**		2650	477	118	>10000
**6**		5400	629	428	>10000
**7**		5335	1238	156	>10000
**8**		1340	96.1	624	94
**9**		1250	115	598	237
**10**		864	85.3	34	97
**11**		729	349	36	336
**12**		1346	215	40	4650
**AAZ**	–	250	12	63	303

* Mean from 3 different assays, errors in the range of ±5–10% of the reported values (data not shown).

a Work by Sławinski et al.^12^.

b This work.

**Table 2.** Inhibition of human (h) CA isoforms hCA I and II and bacterial (*Brucella suis*) enzymes BsuCA1 and BsuCA2 with sulfonamides **13–18**, by a stopped-flow CO_2_ hydrase assay^20^. Acetazolamide (**AAZ**) was used as standard inhibitor.
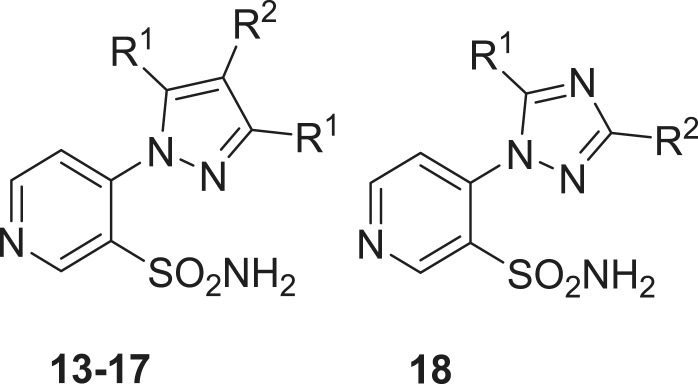

			K_I_ (nM)*			
No	R^1^	R^2^	hCA I^a^	hCA II^a^	BsuCA1^b^	BsuCA2^b^
**13**	Me	H	346	61.4	38	780
**14**	Me	Me	438	58.5	45	884
**15**	Me	Bu	541	76.3	47	853
**16**	Me	CH_2_CH_2_CO_2_Et	397	134	41	706
**17**	Et	H	278	87.1	96	420
**18**	NH_2_	SMe	169	128	112	836
**AAZ**	–	–	250	12	63	303

* Mean from 3 different assays, errors in the range of ±5–10% of the reported values (data not shown);

a Work by Sławinski et al.^12^.

b This work.

The following structure–activity relationship for the inhibition of the two bacterial CAs with sulphonamides **1–18** can be drawn from data of Tables 1 and 2:

- BsuCA1 was rather sensitive to be inhibited by sulphonamides **1–18** investigated here, showing K_I_s ranging between 34 and 624 nM (Table 1). The nature of the ring appended in position 4 of the pyridine sulphonamide and the moieties substituting it were the most important factors influencing enzyme inhibitory properties of these compounds. Thus, the derivatives incorporating the five-membered heterocyclic rings present in **13**–**18** were generally more effective than sulfonamides incorporating substituted piperazines/piperidines **1–12**. For the six-membered ring substituted derivatives, the substitution patterns leading to the most effective inhibitors were 4-chloro/fluorophenyl (**2** and **3**); benzyl (**10** and **12**) and piperonyl (**11**), all these compounds being more effective as BsuCA1 inhibitors compared to the standard inhibitor acetazolamide. For the second subset, only the triazole derivative **18** was slightly less effective as BsuCA1 inhibitor (K_I_ of 112 nM) whereas all pyrazoles **13–17** had K_I_s < 100 nM, in the range of 38–96 nM. The substitution patterns connected with the most effective inhibition were those present in **13–16** (R^1^ a methyl group, and R^2^ being a H, Me, Bu or ethoxycarbonylethyl moiety). For the first subset the least effective inhibitors (**4–9**) incorporated diverse substituents at the 4-phenyl-piperazine moiety, of the type *o*-fluoro-phenyl; 3,4-dichlorophenyl; *o*-methoxyphenyl, 2,5-dimethylphenyl). It is obvious that small modifications in the substitution pattern and nature of the moieties present on the phenyl ring in the 4-arylsubstituted piperazines strongly influence the biological activity.
- BsuCA2 was slightly less sensitive to inhibition with sulphonamides **1–18** compared to BsuCA1, but this behaviour was already reported in previous inhibition studies of these two enzymes^9–11^. Thus, **5–7** did not substantially inhibit this enzyme up to 10 µM concentration of inhibitor within the assay system. Weak BsuCA2 inhibitors were also sulfonamides **1, 3, 4, 9, 11, 12** and **13–18**, with K_I_s in the range of 237 – 4650 nM (Tables 1 and 2). Thus, for this enzyme, the 5-membered ring-substituted derivatives **13–18** were only medium potency – weak inhibitors (in contrast to what observed for the first isoform, BsuCA1, as mentioned above). The most effective BsuCA2 inhibitors were **2, 8** and **10**, with K_I_s in the range 62–97 nM. It may be observed that these three sulfonamides are 3–5 times better BsuCA2 inhibitors compared to acetazolamide, and these are indeed relevant data. As for BsuCA1, small changes in the scaffold of the inhibitor lead to drastic differences of activity. For example, introduction of Cl in the *para* position of the phenyl moiety of **1** led to an increase in the inhibitory power of 13.9 times for the sulphonamide **2**, the best BsuCA2 inhibitor detected so far (Table 1).
- Most of the investigated sulphonamides were weak hCA I and II inhibitors^12^ (Table 1) making them of great interest for more detailed inhibition of growth studies of the pathogen, *ex vivo* and possible also *in vivo*.

## 4. Conclusions

We have investigated in this article the inhibition of the two β-CAs present in the pathogenic bacterium *Brucella suis*, BsuCA1 and BsuCA2, for their inhibition profile with a series of pyridine-3-sulfonamide derivatives incorporating 4-heterocyclic/heteroaryl moieties, originally reported as inhibitors of the tumour-associated human isoforms hCA IX and XII. BsuCA1 was effectively inhibited by these sulphonamides with inhibition constants ranging between 34 and 624 nM. BsuCA2 was less sensitive to these inhibitors, with K_I_s in the range of 62 nM - > 10 µM. The nature of the 4-substituent present on the pyridine ring was the main factor influencing the inhibitory profile against both isoforms, with 4-halogenophenylpiperazin-1-yl and 3,4,5-trisubstituted-pyrazol-1-yl derivatives showing the most effective inhibition. Some of these sulphonamides were most effective bacterial CA than human (h) CA I and II inhibitors, making them selective for the prokaryotic enzymes. Investigation of bacterial CA inhibitors may be relevant for finding antibiotics with a new mechanism of action compared to the clinically used agents for which substantial drug resistance emerged.
